# Understanding the skin blackening phenomenon in Youzhou Dark goats based on the histological characteristics of melanocytes

**DOI:** 10.5455/javar.2024.k865

**Published:** 2024-12-29

**Authors:** Cancan Chen, Jie Li, Xiaoyan Sun, Jing Jiang, Shipeng Lv, Liangjia Liu, Gaofu Wang, Hangxing Ren

**Affiliations:** 1 Chongqing Academy of Animal Sciences, Chongqing, China; 2 Chongqing Engineering Research Center for Goats, Chongqing, China; † These authors contributed equally to this work.

**Keywords:** Youzhou dark goat, Pigmentation, Histology, Transcriptomics, *ASIP*

## Abstract

**Objective:**

The study was conducted to identify the molecular mechanism of the phenotype formation of Youzhou black sheep by histological cytology and transcriptomics.

**Materials and Methods:**

In this study, HE and IHC staining were used to explore the patterns and cytological differences in skin pigment deposition between Youzhou Dark goats and Banjiao goats. In addition, the DEGs related to the black skin phenotype were identified via transcriptomic analyses. Finally, the expression pattern of the agouti signal protein (ASIP) gene in the skin from individuals with different skin color phenotypes was verified by FISH.

**Results:**

The results showed that compared with that on the skin surface of Banjiao goats, melanin deposition on the skin surface of Youzhou Dark goats was abnormally increased. The transcriptomic analyses showed that the expression of the *ASIP* genes decreased significantly in Youzhou Dark goats. FISH confirmed that the expression of the *ASIP* gene in Youzhou Dark goats was significantly lower than that in Banjiao goats.

**Conclusion:**

The present study showed that a decrease in *ASIP* gene expression and an increase in melanin production are important factors associated with skin pigmentation in Youzhou Dark goats.

## Introduction

As the main barrier against the external environment, the skin plays an important role in absorbing ultraviolet rays, regulating body temperature, maintaining body health, and maintaining the appearance characteristics of different varieties [1]. The color of the skin, hair, and eyes is determined by the number, size, composition, and distribution of melanosomes produced by melanocytes [2]. In goats, coat color phenotypes are extremely diverse, but skin color is mostly pink or white. The Youzhou Dark goat, which is a precious local goat resource in southwest China, is the only goat breed with dark skin and white hair; that is, the skin and visible mucosa of this goat are completely black, the hair color is mainly white, and the dorsal line color is black [3]. Interestingly, the researchers found that the meat, beak, skin, bones, and legs of black-bone chicken are all black, and the feathers are white [4]. The black goats in Youzhou only show that the visible skin or mucous membrane is black. Therefore, it is very interesting to analyze this special phenotype.

It is well known that pigmentation is a polygenic trait that is controlled by interactions and quantitative gene effects. The MITF, MC1R, PAX3, and KIT genes are related to the formation of bovine coat color [5]. The ASIP, MC1R, TYR, TYRP1, DCT, PMEL, Cdk5, SLC45A2, and MLANA genes may be related to the regulation of natural coat color development in sheep [6-8]. However, the question of how the deposition of skin pigmentation is controlled has not been resolved. Recent studies have shown that the phenotype of black fur covering the whole body of Minxian black fur sheep is related to the differential expression of OCA2, DCT, TYR, TYRP1, MC1R, and PMEL [9]. In addition, in sheep, the expression levels of BMP4 and noggin [10] in black skin are significantly higher than those in white skin, suggesting that these genes may be related to the regulation of sheep skin color development. Agouti signal protein (ASIP) is one of the key roles in the regulation of hair pigmentation in mammals; the agouti protein is the most prominent and determines the extent of melanogenesis and the type of melanin synthesized [11]. Meanwhile, structural variations in ASIP are the main driver of the differences in coat color among cattle and sheep [12,13]. Therefore, this gene is also a focus of our attention.

Skin pigmentation differs among species at different developmental stages, and the existing evidence cannot explain the molecular basis of the development of the black skin and white hair traits in the Youzhou Dark goat. To clarify the reasons for the unique skin characteristics of Youzhou Dark goats, we analyzed the cytological mechanism of a black character in these goats by means of histocytology and transcriptomics. This study is highly important for understanding the mechanism underlying the black skin phenomenon in Youzhou Dark goats. In addition, the study of black skin characters in the Youzhou Dark goat can provide a theoretical foundation for the cultivation of new breeds of Youzhou Dark goats and the development of clinically useful animal models.

## Materials and Methods

### Animals and ethical approval

The Youzhou Dark and Banjiao goats used in this study were obtained from the Youzhou Dark goat breeding farm in Chongqing, China, which has more than ten years of experience in feeding and managing Youzhou Dark goats and possesses rich and comprehensive genetic resources for this breed. All of the animal experiments performed in this study were approved by the Animal Ethics Committee of Chongqing Academy of Animal Science (approval number: cstc2021jxj180018). In the experiment, three adult Youzhou dark goats (black skin, white hair) and three adult Banjiao goats (white skin, white hair) were selected as a model of skin pigmentation. Skin samples were collected from the left side of the back after slaughter, and the samples were subsequently embedded or stored in liquid nitrogen until they were used for analyses.

### HE staining

After the skin tissue was embedded, the tissue was sliced with a Leica case microtome (Leica, RM2016), thawed with warm water at 42°C, and dried at 60°C. The skin tissue was stained with HE according to the operation manual of the improved HE staining kit (Suolaibao, G1121). After staining, images were taken with an Olympus IX73 inverted fluorescence microscope imaging system. The criteria for HE-stained cells were a blue nucleus and red cytoplasm. The slide images were analyzed using ImageJ software.

### Transmission electron microscopy

Skin tissue samples were prefixed with 3% glutaraldehyde and subsequently postfixed in 1% osmium tetroxide, dehydrated in a series of acetone, infiltrated with Epox 812 for a long period, and embedded. The sections were stained with methylene blue, and ultrathin sections were cut with a diamond knife and stained with uranyl acetate and lead citrate. Sections were examined with a JEM-1400-FLASH transmission electron microscope.

### Transcriptome data analysis

Total RNA was extracted from goat skin by the TRIzol method. The purity of the extracted RNA was detected by 1.5% agarose gel electrophoresis, and the quality was detected by a NanoDrop 2000 instrument. For samples that passed quality testing, sequencing libraries were constructed by Beijing Novogene Biological Co., Ltd., and Illumina HiSeqTM2500 sequencing was carried out for libraries that passed quality testing. A large amount of skin transcriptome data from goats with different skin colors was obtained, and annotation information was obtained by comparison to the KEGG and GO databases. GO enrichment analysis annotates factors in terms of three categories: cell component (CC), molecular function (MF), and biological process (BP). KEGG enrichment analysis annotates genes into corresponding signaling pathways for subsequent analysis. In these analyses, *p < 0.05 was set as the critical value for statistical significance.

### In situ hybridization

The ASIP (BAC-ONT6666.1) probe was synthesized by Wuhan Servicebio Technology Co., Ltd., and the probe sequence was (5’-3’) TTATG CAGCA GAGG AAG ATTGTGT. The experiment was carried out with the paraffin section SweAMI-DAB chromogenic *in situ* hybridization platform. Goat skin tissue was fixed with 4% paraformaldehyde for 24 hours and then embedded in paraffin. Protease K was added for digestion for 5 min, after which the section was hybridized overnight at 42°C with a hybridization solution containing the ASIP probe. The cells were incubated for 30 min at room temperature with serum to block nonspecific staining, and then anti-digoxin-labeled peroxidase was added. The sections were incubated with DAPI dye solution in the dark for 8 min, rinsed, and sealed with anti-fluorescence quenching tablets. Finally, the slices were observed, and images were acquired under a Nikon fluorescence microscope. The criterion was that the nucleus was stained both blue and red.

### Immunohistochemistry

After the skin tissue was embedded, the tissue was sliced with a Leica case microtome (Leica, RM2016). Tissue sections (4–5 μM) were dewaxed with xylene and rehydrated with gradient alcohol, and immunohistochemistry experiments were carried out according to the instructions of the immunohistochemical kit (Bioss, IHC001). The rehydrated slices were treated with antigen repair solution in boiling water for 15 min and sealed with a sealing solution containing 5% horse serum for 1 h, after which the sections were incubated overnight with the primary antibody. The MITF antibody was obtained from Abcam (ab140606, 1:100 dilution), the Mlana antibody was obtained from CST (64718, 1:100 dilution), the S100b antibody was obtained from CST (42397, 1:500 dilution), the SOX10 antibody was obtained from CST (69661, 1:100 dilution), the TYR antibody was obtained from Abcam (ab180753, 1:100 dilution), the TYRP1 antibody was obtained from Abcam (ab235447, 1:100 dilution), and the DCT antibody was obtained from Abcam (ab221144, 1:100 dilution). After overnight incubation with the primary antibody, the sections were washed with PBS and labeled with a fluorescent goat anti-rabbit IgG secondary antibody. Finally, images were acquired with an Olympus IX73 inverted fluorescence microscope and imaging system. The slide images were analyzed using ImageJ software.

### Statistical analysis

All experiments were repeated at least three times. The data were statistically analyzed according to the SPSS 20.0 K-S normal distribution. All the data are shown in the form of bar charts as the mean ± standard error of the mean (SEM). Differences with **p < 0.01 were regarded as statistically significant and very significant.

## Results

**Figure 1.**
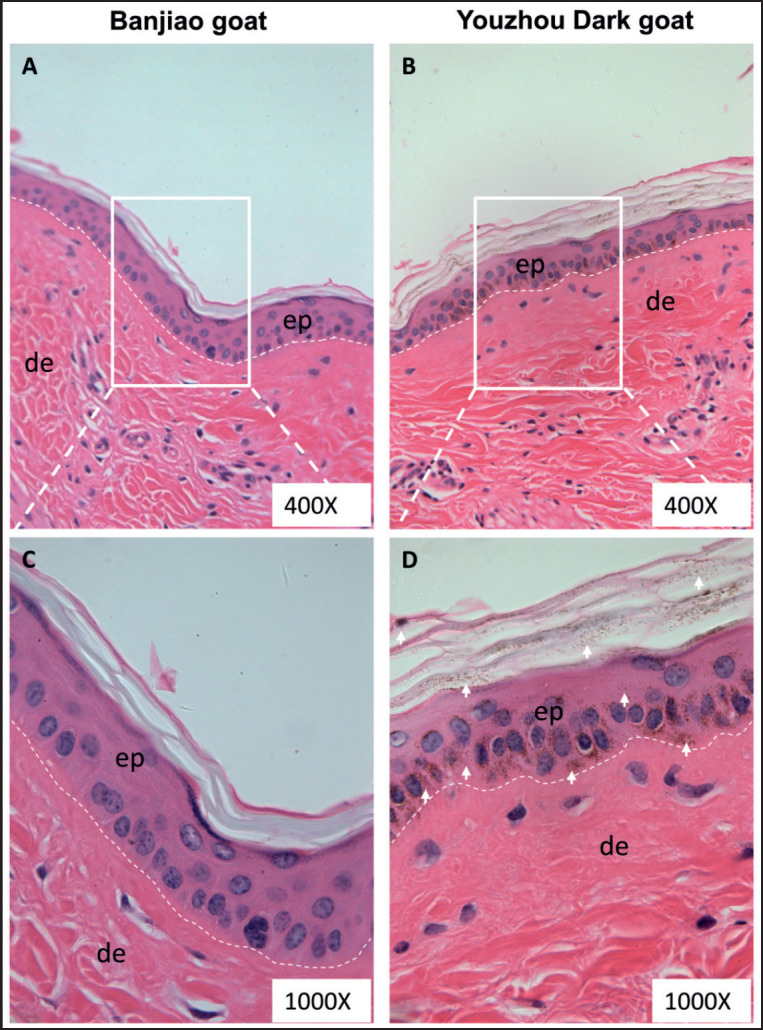
Distribution of melanin in the epidermis. (A, C) Banjiao goat skin sections stained with HE at various magnifications. (B, D) Youzhou Dark goat skin sections stained with HE at various magnifications. Note: the arrow indicates melanin, ep. Epidermis, de. Dermis; the same applies below.

Melanin is widely distributed on the epidermis of Youzhou Dark goats HE staining clearly revealed differences in the distribution of melanin granules among the breeds, with no significant deposition of melanin particles on the epidermis of Banjiao goats with the white skin phenotype (Figure 1A, C). In contrast, many melanin particles were distributed from the basal layer to the stratum corneum of the epidermis in Youzhou Dark goats. In detail, there is a lot of melanin deposition in the basal layer of the skin epidermis (Figure 1B, D). Figure 1 shows that the melanin content in the skin samples of Youzhou black goats was significantly greater than that in the skin samples of Banjiao goats.

**Figure 2.**
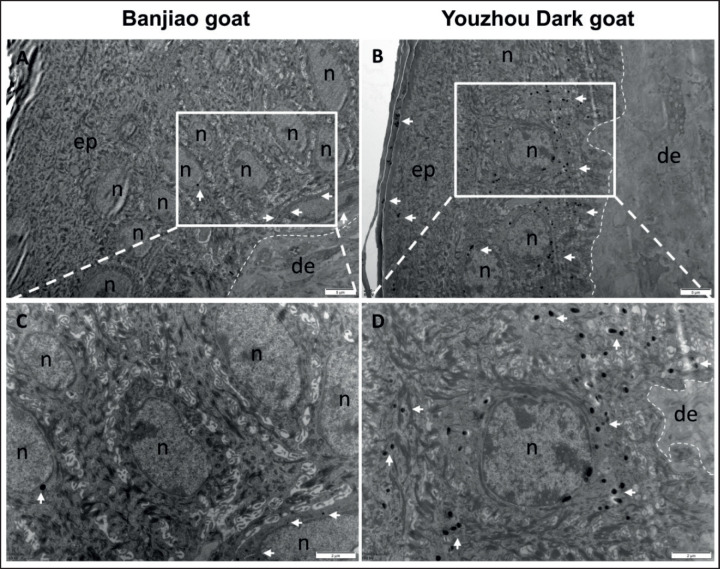
Comparison of goat skin tissue histology via transmission electron microscopy. (A, C) Transmission electron microscope slices of Banjiao goat skin at different resolutions; (B, D) transmission electron microscope slices of Youzhou Dark goat skin at different resolutions. Note: n. Nucleus.

The results of transmission electron microscopy showed that there was no obvious distribution of melanosomes in the epidermis of Banjiao goat skin (Figure 2A, C), whereas there were many melanosomes in the cytoplasm of the epidermis of Youzhou Dark goat skin; moreover, the volume of these melanosomes was significantly greater than that of melanosomes in white skin (Figure 2B, D). Melanosomes at different stages can be seen in the basal layer and prickle layer cells, and the number is significantly higher than that in the skin of Banjiao goats, which indicates that melanocytes have the active function of synthesizing melanin, and the transport of melanosomes to keratinocytes is increased. The number of melanocytes was counted via transmission electron microscopy. Melanocytes of different stages and larger sizes were found in the basal layer of Youzhou Dark goat skin. The above results confirmed that the black skin phenotype of the Youzhou Dark goats was due to the formation and deposition of a large number of melanosomes on the skin and that the number, size, and distribution of melanosomes in the skin of the Youzhou Dark goats were much greater than those in the skin of the Banjiao goats.

### Transcriptome analysis

**Figure 3.**
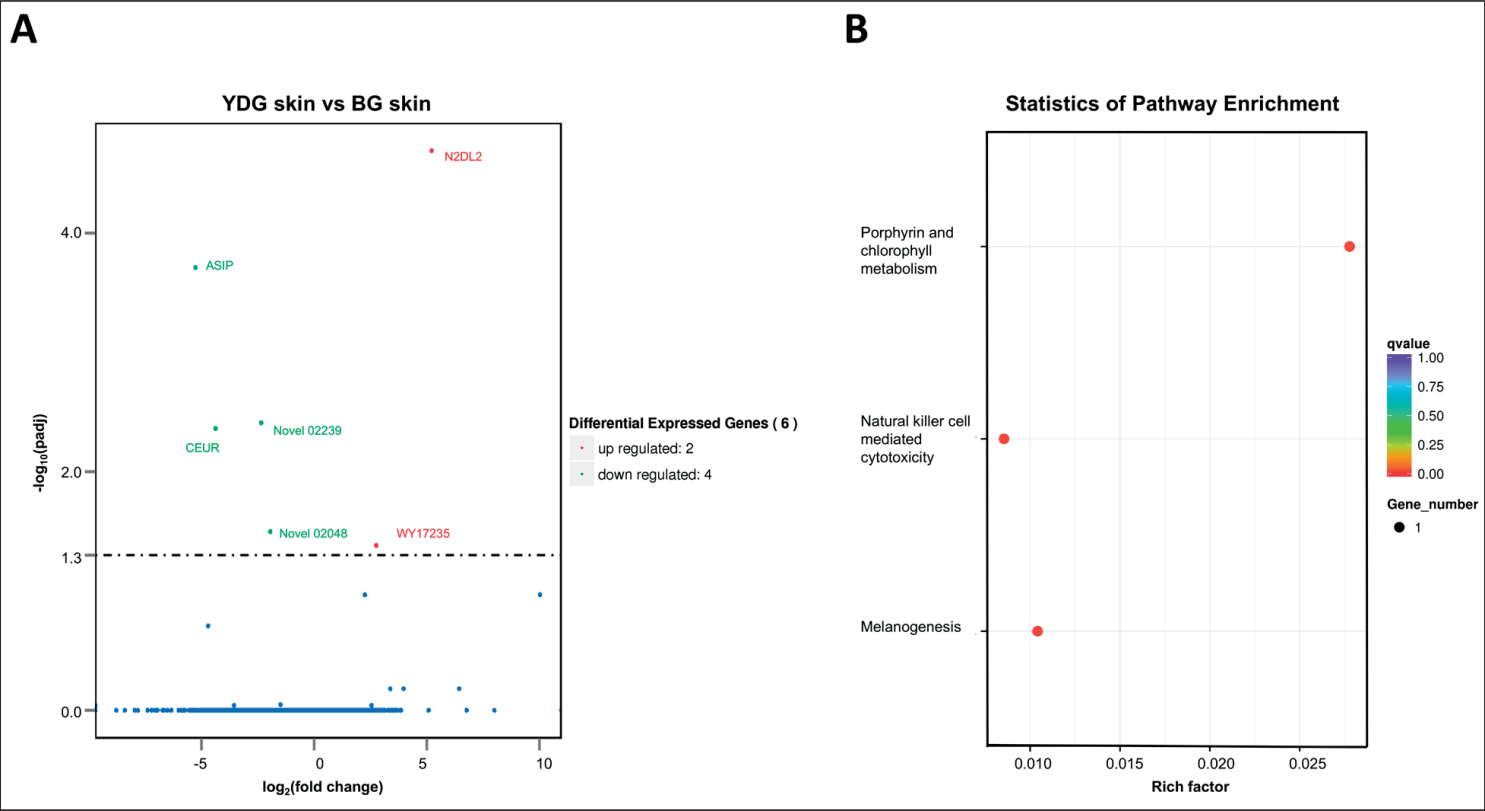
Transcriptome sequencing revealed the gene expression characteristics associated with skin color development in Youzhou Dark goats and Banjiao goats. (A) Volcano plot of DEGs. Transcriptome analysis revealed the top 6 genes related to skin color. The thresholds used to define DEGs were |log2 (fold change) | > 1 and padj < 0.05. (B) Scatter plot of the KEGG pathways enriched in DEGs.

The expression of these genes in adult goat skin was detected at the transcript level. As shown in Figure 3A, there were six differentially expressed genes (DEGs) in the skin of Youzhou Dark goats and Banjiao goats, with three annotated genes, two new genes, and one unannotated gene. The DEGs were subjected to KEGG pathway enrichment analysis (https://www.genome.jp/kegg1.html) (Fig. 3B). The results showed that one DEG in the melanin synthesis pathway was *ASIP* (Table 1). Therefore, the phenotype of black skin traits in Youzhou Dark goats may be regulated by *ASIP* expression.

ASIP *in situ* hybridization

**Figure 4.**
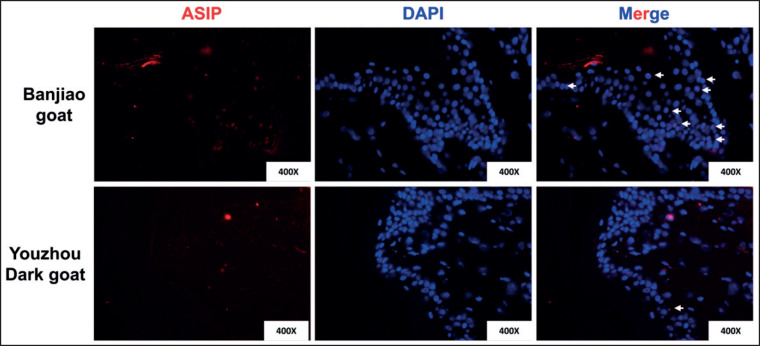
*In situ* hybridization observations. Red indicates positively stained cells. Magnification: 400×.

*In situ* hybridization with ASIP specifically labeled the basal layers of the skin of both Banjiao goats and Youzhou Dark goats (Figure 4). The Banjiao goat and Youzhou Dark goat both express the ASIP gene in keratinocytes in the basal layer of skin. The expression of ASIP in the skin of the Banjiao goats was significantly greater than that in the skin of the Youzhou Dark goats, and the high expression of ASIP was positively correlated with the white phenotype, which further confirmed that the decrease in ASIP expression led to the phenotype of the Youzhou Dark goats.

**Figure 5.**
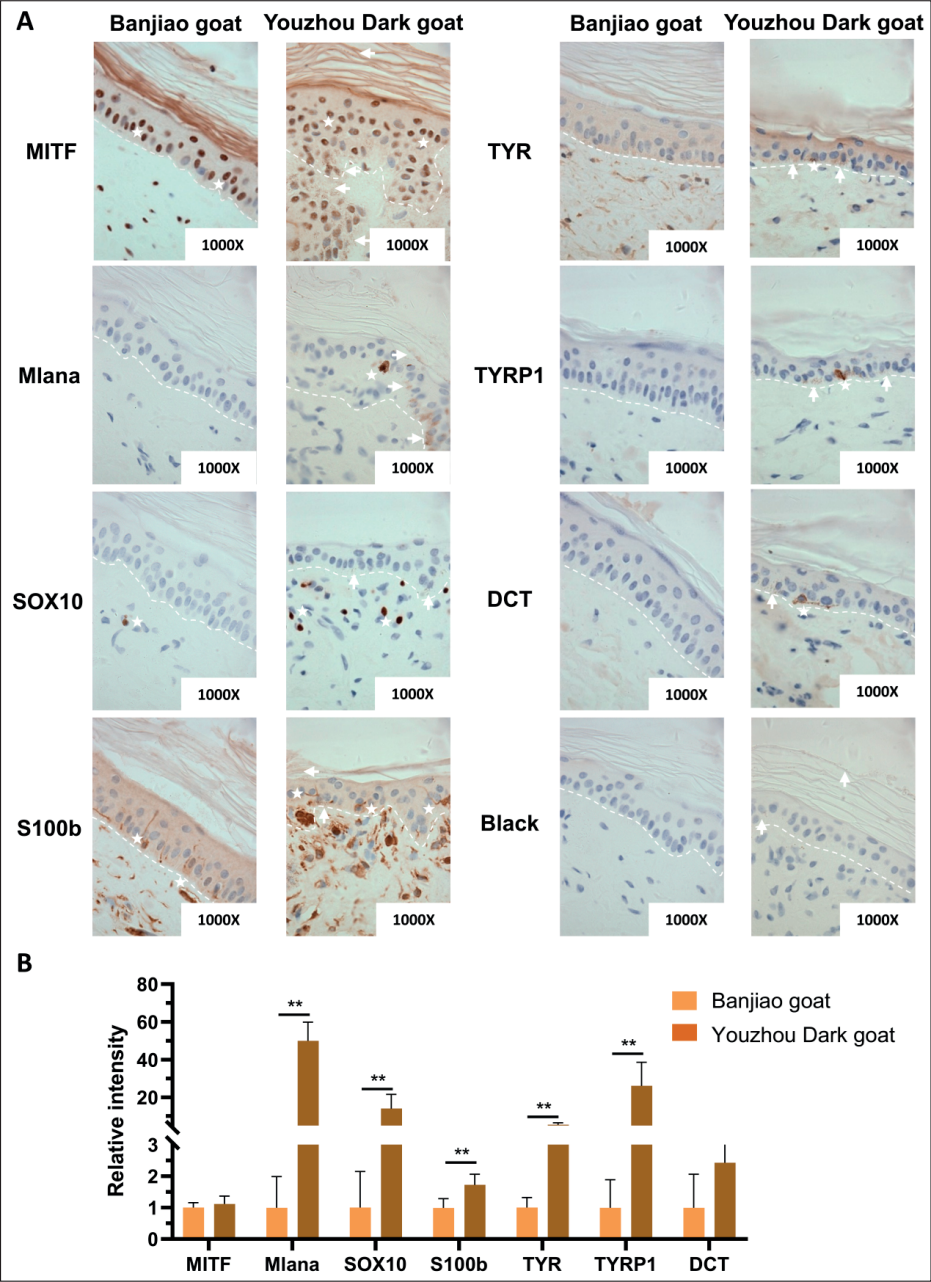
The activity of skin melanocytes and the expression levels of related proteins were detected via immunohistochemistry. (A) Immunohistochemical sections of goat skin; magnification: 1000×; (B) Quantification of the data.

**Figure 6.**
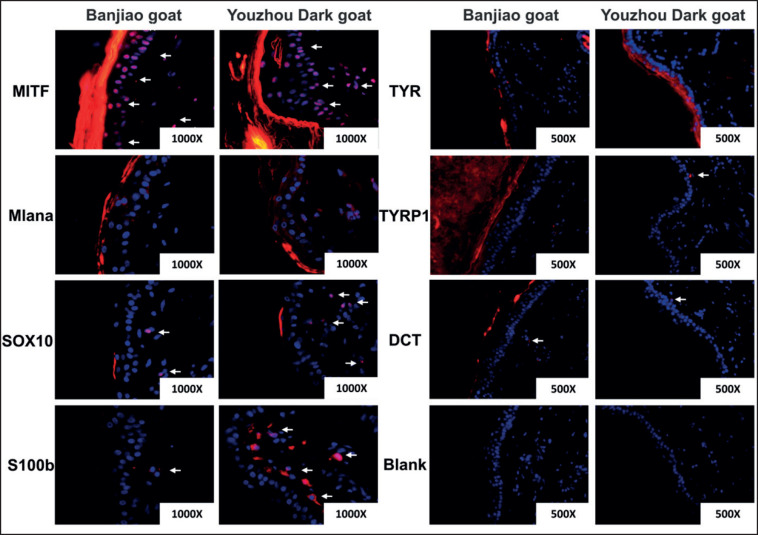
Immunofluorescence was used to detect the activity of skin melanocytes and the expression levels of related proteins. MITF, Mlana, SOX10 and S100b, magnification: 1000×. TYR, TYRP1, DCT and Blank, magnification: 500×

The activity of melanocytes in the skin is greater in Youzhou Dark goats than in Banjiao goats. Immunohistochemistry and immunofluorescence analyses indicated that there was no significant difference in MITF or DCT expression between Banjiao goat skin and Youzhou Dark goat skin (*p* > 0.05); however, the expression levels of Mlana, SOX10, S100b, TYR, and TYRP1 were significantly higher in the skin of Youzhou Dark goats than in the skin of Banjiao goats, as shown in Figure 5 (*p* < 0.01). SOX10 is a melanocyte-specific nuclear marker that can be used to count melanocytes. S100B, TYR, TYRP1, and DCT are closely related to melanocyte synthesis and can be used to determine melanocyte activity. The immunofluorescence results were consistent with these findings (Figure 6). The above results showed that the number and activity of melanocytes in the skin of Youzhou Dark goats were greater than those in the skin of Banjiao goats.

**Table 1.** KEGG annotation table of DEGs.

Gene symbol	Expression	p value	KEGG Pathway
ASIP	down	1.75E-08	Melanogenesis
CERU	down	7.80E-07	Porphyrin and chlorophyll metabolism
Novel02239	down	5.23E-07	Unannotated
Novel02048	down	7.14E-06	Unannotated
N2DL2	up	9.16E-10	Natural killer cell mediated cytotoxicity
WY17235	up	1.12E-05	Unannotated

## Discussion

Melanin is the main pigment in mammalian skin, hair, and eyes. The molecular regulation of melanogenesis is complex; tyrosinase is the necessary and rate-limiting enzyme for melanin production, and its activity strictly requires a neutral pH [14]. Related studies have confirmed that the melanosome is a specialized intracellular organelle that produces and stores melanin in melanocytes. The structure and function of melanosomes are determined by a group of resident transmembrane proteins. Melanosomes gradually form and mature in melanocytes (stages I-IV) and are subsequently transported from the perinuclear region to the cell periphery along the cytoskeleton. The melanosomes are transferred to adjacent keratinocytes through unknown mechanisms and transported to the perinuclear region of the keratinocytes [15,16]. To clarify the pattern of skin pigment deposition in Youzhou Dark goats, transmission electron microscopy was performed, and melanocytes were identified according to the distribution pattern of melanosomes. There were many melanin granules in the epidermis of Youzhou Dark goats; the melanosomes were large and clustered, and the number of melanocytes was significantly greater than that in Banjiao goats, with obvious histological differences. This finding is similar to the research of Shi et al. [9] and Wang et al. [17]. Melanosomes at different stages in Youzhou Dark goats can be seen in the basal layer and prickle layer cells, and the number is significantly higher than that in the skin of Banjiao goats, which indicates that melanocytes have the active function of synthesizing melanin and increasing the transport of melanosomes to keratinocytes.

Melanosomes produced by melanocytes are regulated by many genes and signaling pathways, and related regulatory genes can also be used as molecular markers of melanocytes. For example, MITF, an important regulator, regulates melanocyte development, melanogenesis, and survival [18]; MITF also modulates melanocyte differentiation [19]. Melan-A is one of the most common markers used to detect the presence and distribution of melanocytes [20]. S-100 is the most sensitive marker of melanocytopathy [21]. SOX10 plays an important role in the synthesis and normal functional maintenance of melanocytes. SOX10 strongly affects the development of melanocytes, and a decrease in the SOX10 gene leads to a decrease in melanoblasts [22,23]. In addition, SOX10 immunostaining can be used to quantify melanocyte density and evaluate the size of melanocyte nuclei [24]. Previous studies have confirmed that skin color and coat color are related to the activity of melanocytes, the number, size, and shape of melanosomes, and the degree of blackening [25]. According to our research, the number and activity of melanocytes in the skin of Youzhou Dark goats were greater than those in the skin of Banjiao goats. Taken together, these findings show that the pigmentation of Youzhou Dark goat skin is due to the synergistic effect of the number and activity of melanocytes.

To understand the molecular mechanism of skin color formation in Youzhou Dark goats and clarify the genes that play important roles in skin pigmentation, the DEGs between the skin of Banjiao goats and Youzhou Dark goats were analyzed via transcriptome sequencing. Among the genes related to melanin synthesis, only *ASIP* expression was significantly decreased. *ASIP *and other genes are the main molecular basis of abnormal pigmentation resulting from animal hair color and skin color [13, 26], and structural modification of the *ASIP* gene can lead to an albino phenotype in different species [27]. It has been found that in white buffalo skin, insertion of a 2809 bp *LINE-1* sequence in the *ASIP* gene leads to a 10-fold increase in the transcription of the *ASIP* gene, causing pigment deposition deficiency leading to white skin and hair follicles [12,28]. Similarly, variation in the *ASIP* sequence is the reason for the darkening of coat color in specific parts of Nellore cattle [26]. The tandem repeat sequence of the *ASIP* locus in Merino sheep leads to a dominant white hair phenotype [29]. In addition, there is an *ITCH-ASIP* gene fusion, which can cause increased transcription levels, thus leading to feather color lightening in quail with a fawn/beige phenotype [30]. In summary, the increased transcription and expression of the *ASIP* gene results in a white phenotype. Consistent with this, we determined that the transcription level of *ASIP *was lower in Youzhou Dark goats than in white Banjiao goats, representing a potential cause of skin pigmentation in Youzhou Dark goats.

The basic mechanisms involved in pigmentation, including the dependency on the* ASIP *gene and the complexity of transcription and expression changes in different species, may be related to age and tissue type in many animals. This study showed that both Youzhou Dark goats and Banjiao goats expressed ASIP in the basal layer of the skin, and the transcriptome analysis showed that *ASIP* was the only pigment pathway gene expressed differently between Youzhou Dark goats and Banjiao goats. We proved that the main cause of melanin deposition in adult Youzhou Dark goats was *ASIP* gene expression. It is speculated that the early termination of *ASIP *translation may lead to decreased expression of *ASIP*, while the expression of the melanin-related proteins SOX10, TYR, TYRP1, and DCT is activated, thus inducing melanocytes to produce many melanosomes. Melanosomes transfer melanin to surrounding keratinocytes through the action of transfer-related genes, and many melanin granules are also deposited on the stratum corneum of the skin through natural metabolism and apoptosis. Our research provides new insight into this phenomenon; that is, the pigmentation of Youzhou Dark goat skin is not only the result of a cascade reaction caused by a change in *ASIP* expression but also closely related to an increase in melanocyte number.

## Conclusion

We analyzed the mechanism of pigment deposition in goat skin of different colors via histological methods. The number of skin melanocytes and melanosomes increased significantly, the activity of melanocytes was significantly greater, and the expression of *ASIP* was significantly lower in Youzhou Dark goats than in Banjiao goats. A significant decrease in *ASIP* expression is the main reason for the increased pigmentation. However, the increase in the number of melanocytes is also an important reason for the dark phenotype of Youzhou Dark goat skin. Our results preliminarily reveal the skin pigment deposition pattern of Youzhou Dark goats. However, further validation is needed to fully understand whether the *ASIP* gene is the determining gene that leads to its dark skin phenotype.

## List of abbreviations

BP, Biological process; CC, Cell component; DEGs, Differentially expressed genes; DE, Dermis; EP, Epidermis; FISH, Fluorescence *in situ* hybridization; GO, Gene ontology; HE staining, Hematoxylin-eosin staining; KEGG, Kyoto encyclopedia of genes and genomes; MF, Molecular function; N, nucleus.
